# PD22 Economic Evaluation Of Polygenic Risk Scores In Clinical Practice: A Systematic Review

**DOI:** 10.1017/S0266462324002861

**Published:** 2025-01-07

**Authors:** Claudia Isonne, Jessica Iera, Francesco Pierri, Giuseppe Migliara, Valentina Baccolini, Antonio Sciurti, Leonardo Maria Siena, Immacolata Leone, Carolina Marzuillo, Paolo Villari

## Abstract

**Introduction:**

Implementation of polygenic risk scores (PRS) may improve disease prevention and management, but its benefits and costs are still to be quantified. The review aimed to summarize evidence on the economic evaluation of PRS, exploring how their use impacts healthcare resource utilization, cost outcomes, and overall cost-effectiveness in order to help guide healthcare decision-making and resource allocation.

**Methods:**

The PubMed, Scopus, and Web of Science databases were searched. The search terms were constructed to identify full economic evaluations of healthcare programs containing PRS aimed at assessing the risk of diseases or other health outcomes in any target population. The search strategy was adapted to fit each database’s research criteria. The literature search was supplemented by scanning the reference lists of retrieved articles. No language or date restriction was applied. The database search was rerun just before the final analysis. This research was supported by the European Commission and the Ministry for Universities and Research under the National Recovery and Resilience Plan (M4C2-I1.3 Project PE_00000019 “HEAL ITALIA”).

**Results:**

Of the 1,891 articles screened, seven economic evaluations were included. They were conducted between 2020 and 2022 in Asia (n=1), Australia (n=1), Europe (n=2), and the USA (n=3). The field of application was ophthalmology (n=2), oncology (n=1), cardiovascular (n=2), nephrology (n=1), and endocrinology (n=1). The time horizon was lifetime (57%) or between five and 40 years (43%). The most frequent viewpoint was the health system perspective (n=5). The target populations included healthy people or individuals at risk for a specific disease. Performing PRS tests was found to be a dominant strategy, with lower costs and better health outcomes in all studies.

**Conclusions:**

Although the integration of PRS in clinical practice seems to be a cost-effective strategy, the small number of studies available and the heterogeneity in the field of application and the target populations limits the generalizability of results. A health technology assessment approach could be useful for summarizing the evidence on all domains of PRS implementation.

